# *N*-(2,6-Dichloro­phen­yl)-4-methyl­benzamide

**DOI:** 10.1107/S1600536811028935

**Published:** 2011-07-23

**Authors:** Vinola Z. Rodrigues, Sabine Foro, B. Thimme Gowda

**Affiliations:** a Department of Chemistry, Mangalore University, Mangalagangotri 574199, Mangalore, India; b Institute of Materials Science, Darmstadt University of Technology, Petersenstrasse 23, D-64287, Darmstadt, Germany

## Abstract

In the title compound, C_14_H_11_Cl_2_NO, the two aromatic rings are nearly orthogonal to each other [dihedral angle 79.7 (1)°], while the central amide core –NH—C(=O)– is nearly coplanar with the benzoyl ring [N—C—C—C torsion angles = −5.5 (3) and 1772. (2)°]. In the crystal, inter­molecular N—H⋯O hydrogen bonds link the mol­ecules into *C*(4) chains propagating in [001].

## Related literature

For our studies on the effects of substituents on the structures of *N*-(ar­yl)-amides, see: Bhat & Gowda (2000 ▶); Gowda *et al.* (2006 ▶, 2009 ▶), on *N*-(ar­yl)-methane­sulfonamides, see: Jayalakshmi & Gowda (2004 ▶) and on *N*-(ar­yl)-aryl­sulfonamides, see: Gowda *et al.* (2005 ▶).
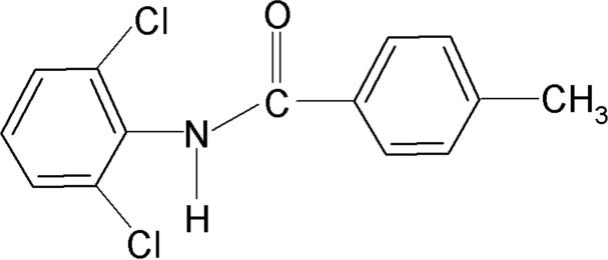


## Experimental

### 

#### Crystal data

C_14_H_11_Cl_2_NO
*M* *_r_* = 280.14
Tetragonal, 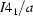

*a* = 16.4706 (8) Å
*c* = 19.8709 (9) Å
*V* = 5390.6 (4) Å^3^
*Z* = 16
Mo *K*α radiation
μ = 0.47 mm^−1^
*T* = 293 K
0.48 × 0.34 × 0.14 mm

#### Data collection

Oxford Diffraction Xcalibur diffractometer with Sapphire CCD detector
Absorption correction: multi-scan (*CrysAlis RED*; Oxford Diffraction, 2009 ▶) *T*_min_ = 0.807, *T*_max_ = 0.937
5852 measured reflections
2752 independent reflections
1701 reflections with *I* > 2σ(*I*)
*R*_int_ = 0.021

#### Refinement

*R*[*F* ^2^ > 2σ(*F* ^2^)] = 0.042
*wR*(*F* ^2^) = 0.120
*S* = 1.01
2752 reflections
166 parameters
1 restraint
H atoms treated by a mixture of independent and constrained refinement
Δρ_max_ = 0.23 e Å^−3^
Δρ_min_ = −0.21 e Å^−3^

### 

Data collection: *CrysAlis CCD* (Oxford Diffraction, 2009 ▶); cell refinement: *CrysAlis RED* (Oxford Diffraction, 2009 ▶); data reduction: *CrysAlis RED*; program(s) used to solve structure: *SHELXS97* (Sheldrick, 2008 ▶); program(s) used to refine structure: *SHELXL97* (Sheldrick, 2008 ▶); molecular graphics: *PLATON* (Spek, 2009 ▶); software used to prepare material for publication: *SHELXL97*.

## Supplementary Material

Crystal structure: contains datablock(s) I, global. DOI: 10.1107/S1600536811028935/bt6816sup1.cif

Structure factors: contains datablock(s) I. DOI: 10.1107/S1600536811028935/bt6816Isup2.hkl

Supplementary material file. DOI: 10.1107/S1600536811028935/bt6816Isup3.cml

Additional supplementary materials:  crystallographic information; 3D view; checkCIF report

## Figures and Tables

**Table 1:** Hydrogen-bond geometry (Å, °)

*D*—H⋯*A*	*D*—H	H⋯*A*	*D*⋯*A*	*D*—H⋯*A*
N1—H1*N*⋯O1^i^	0.82 (2)	2.08 (2)	2.878 (2)	164 (2)

Symmetry code: (i) 
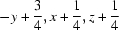
.

**Fig. 1.**
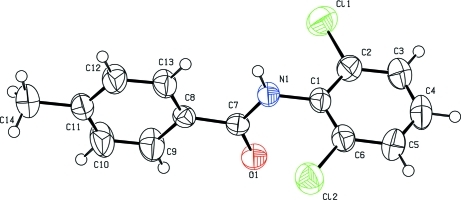
Molecular structure of (I) showing the atom labelling scheme. Displacement ellipsoids are drawn at the 50% probability level and H atoms are represented as small spheres of arbitrary radii.

**Fig. 2.**
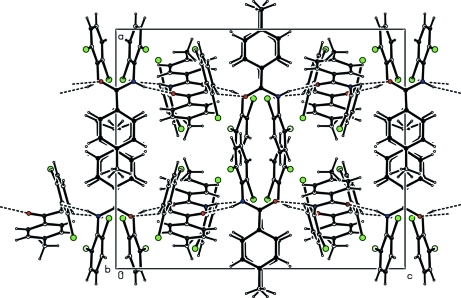
Molecular packing of the title compound with hydrogen bonding shown as dashed lines.

## supplementary crystallographic information

### Comment

The amide moiety is an important constituent of many biologically significant compounds. As part of our studies on the effects of ring and side chain substitutions on the structures of *N*-(aryl)-amides (Bhat & Gowda, 2000; Gowda *et al.*, 2006, 2009), *N*-(aryl)-methanesulfonamides (Jayalakshmi & Gowda, 2004) and *N*-(aryl)-arylsulfonamides (Gowda *et al.*, 2005), the crystal structure of *N*-(2,6-dichlorophenyl)-4-methylbenzamide (I) has been determined (Fig. 1). The conformations of the N—H and C=O bonds in the amide segment of the structure are *anti* to each other, similar to that observed in *N*-(2,6-dimethylphenyl)-4-methylbenzamide (II) (Gowda *et al.*, 2009) and other benzanilides, with similar bond parameters.

The two aromatic rings in the structure make the dihedral angle of 79.7 (1)°, compared to the value of 78.8 (1)° in (II). The central amide core –NH—C(=O)– is nearly coplanar with the benzoyl ring. The orientation of the anilino ring with respect to the amide core are given by the torsion angles, C2—C1—N1—C7 = 105.8 (3)° and C6—C1—N1—C7 = -74.5 (3)°.

Part of the crystal structure of (I), showing the formation of hydrogen- bonded layered chains (Table 1) running along *b* axis is shown in Fig.2.

### Experimental

The title compound was prepared by the method described by Gowda *et al.* (2009). The purity of the compound was checked and characterized by recording its infrared and NMR spectra.

Needle-like colourless single crystals of the title compound were obtained by slow evaporation from an ethanol solution of the compound (0.5 g in about 30 ml of ethanol) at room temperature.

### Refinement

The H atom of the NH group was located in a difference map and later restrained to N—H = 0.86 (2) %A. The other H atoms were positioned with idealized geometry using a riding with the aromatic C—H = 0.93Å and the methyl C—H = 0.96 Å. All H atoms were refined with isotropic displacement parameters set to 1.2 times of the *U*_eq_ of the parent atom.

### Crystal data

**Table:**

C_14_H_11_Cl_2_NO	*D*_x_ = 1.381 Mg m^−^^3^
*M**_r_* = 280.14	Mo *K*α radiation, λ = 0.71073 Å
Tetragonal, *I*4_1_/*a*	Cell parameters from 1773 reflections
Hall symbol: -I 4ad	θ = 2.8–27.9°
*a* = 16.4706 (8) Å	µ = 0.47 mm^−^^1^
*c* = 19.8709 (9) Å	*T* = 293 K
*V* = 5390.6 (4) Å^3^	Prism, colourless
*Z* = 16	0.48 × 0.34 × 0.14 mm
*F*(000) = 2304	

### Data collection

**Table:**

Oxford Diffraction Xcalibur diffractometer with Sapphire CCD detector	2752 independent reflections
Radiation source: fine-focus sealed tube	1701 reflections with *I* > 2σ(*I*)
graphite	*R*_int_ = 0.021
Rotation method data acquisition using ω scans.	θ_max_ = 26.4°, θ_min_ = 3.0°
Absorption correction: multi-scan (*CrysAlis RED*; Oxford Diffraction, 2009)	*h* = −12→17
*T*_min_ = 0.807, *T*_max_ = 0.937	*k* = −17→20
5852 measured reflections	*l* = −10→24

### Refinement

**Table:**

Refinement on *F*^2^	Primary atom site location: structure-invariant direct methods
Least-squares matrix: full	Secondary atom site location: difference Fourier map
*R*[*F*^2^ > 2σ(*F*^2^)] = 0.042	Hydrogen site location: inferred from neighbouring sites
*wR*(*F*^2^) = 0.120	H atoms treated by a mixture of independent and constrained refinement
*S* = 1.01	*w* = 1/[σ^2^(*F*_o_^2^) + (0.0618*P*)^2^ + 1.1095*P*] where *P* = (*F*_o_^2^ + 2*F*_c_^2^)/3
2752 reflections	(Δ/σ)_max_ = 0.001
166 parameters	Δρ_max_ = 0.23 e Å^−^^3^
1 restraint	Δρ_min_ = −0.21 e Å^−^^3^

### Special details

**Table:**

Geometry. All e.s.d.'s (except the e.s.d. in the dihedral angle between two l.s. planes) are estimated using the full covariance matrix. The cell e.s.d.'s are taken into account individually in the estimation of e.s.d.'s in distances, angles and torsion angles; correlations between e.s.d.'s in cell parameters are only used when they are defined by crystal symmetry. An approximate (isotropic) treatment of cell e.s.d.'s is used for estimating e.s.d.'s involving l.s. planes.
Refinement. Refinement of *F*^2^ against ALL reflections. The weighted *R*-factor *wR* and goodness of fit *S* are based on *F*^2^, conventional *R*-factors *R* are based on *F*, with *F* set to zero for negative *F*^2^. The threshold expression of *F*^2^ > σ(*F*^2^) is used only for calculating *R*-factors(gt) *etc*. and is not relevant to the choice of reflections for refinement. *R*-factors based on *F*^2^ are statistically about twice as large as those based on *F*, and *R*- factors based on ALL data will be even larger.

### Fractional atomic coordinates and isotropic or equivalent isotropic displacement parameters (Å^2^)

**Table:**

	*x*	*y*	*z*	*U*_iso_*/*U*_eq_	
C1	0.14058 (13)	0.52556 (15)	0.06376 (10)	0.0437 (5)	
C2	0.07328 (15)	0.48135 (16)	0.08354 (12)	0.0542 (6)	
C3	−0.00303 (16)	0.51539 (19)	0.08617 (14)	0.0686 (8)	
H3	−0.0474	0.4849	0.1002	0.082*	
C4	−0.01236 (17)	0.5952 (2)	0.06771 (14)	0.0707 (8)	
H4	−0.0638	0.6186	0.0686	0.085*	
C5	0.05259 (16)	0.64076 (17)	0.04806 (14)	0.0630 (7)	
H5	0.0455	0.6947	0.0356	0.076*	
C6	0.12874 (14)	0.60634 (16)	0.04678 (11)	0.0505 (6)	
C7	0.25465 (13)	0.47064 (13)	0.00212 (10)	0.0397 (5)	
C8	0.33829 (13)	0.43726 (13)	0.00489 (9)	0.0388 (5)	
C9	0.37543 (16)	0.4135 (2)	−0.05361 (12)	0.0726 (9)	
H9	0.3472	0.4172	−0.0940	0.087*	
C10	0.45324 (17)	0.3845 (2)	−0.05353 (13)	0.0792 (9)	
H10	0.4766	0.3692	−0.0942	0.095*	
C11	0.49760 (14)	0.37724 (14)	0.00367 (12)	0.0497 (6)	
C12	0.46104 (15)	0.40214 (18)	0.06207 (12)	0.0655 (8)	
H12	0.4899	0.3992	0.1022	0.079*	
C13	0.38295 (15)	0.43136 (17)	0.06293 (11)	0.0600 (7)	
H13	0.3600	0.4474	0.1036	0.072*	
C14	0.58324 (16)	0.34561 (19)	0.00401 (16)	0.0713 (8)	
H14A	0.6167	0.3796	−0.0238	0.086*	
H14B	0.5839	0.2912	−0.0132	0.086*	
H14C	0.6038	0.3459	0.0492	0.086*	
N1	0.21884 (11)	0.49001 (12)	0.06109 (8)	0.0462 (5)	
H1N	0.2409 (14)	0.4795 (15)	0.0972 (9)	0.055*	
O1	0.21852 (10)	0.48106 (11)	−0.05131 (7)	0.0548 (5)	
Cl1	0.08438 (5)	0.37957 (5)	0.10321 (4)	0.0777 (3)	
Cl2	0.21062 (4)	0.66582 (4)	0.02405 (4)	0.0717 (3)	

### Atomic displacement parameters (Å^2^)

**Table:**

	*U*^11^	*U*^22^	*U*^33^	*U*^12^	*U*^13^	*U*^23^
C1	0.0379 (13)	0.0624 (16)	0.0309 (11)	0.0084 (11)	−0.0007 (9)	−0.0048 (10)
C2	0.0483 (15)	0.0640 (16)	0.0502 (14)	0.0043 (12)	−0.0027 (11)	0.0034 (11)
C3	0.0425 (15)	0.082 (2)	0.0817 (19)	0.0023 (14)	0.0067 (13)	0.0058 (15)
C4	0.0432 (16)	0.086 (2)	0.083 (2)	0.0163 (15)	0.0036 (14)	−0.0081 (16)
C5	0.0530 (16)	0.0590 (17)	0.0770 (18)	0.0150 (13)	−0.0009 (13)	−0.0057 (14)
C6	0.0438 (14)	0.0612 (16)	0.0465 (13)	0.0046 (11)	0.0013 (10)	−0.0056 (11)
C7	0.0430 (12)	0.0448 (13)	0.0315 (11)	0.0010 (10)	−0.0003 (9)	−0.0002 (9)
C8	0.0400 (12)	0.0426 (12)	0.0337 (11)	−0.0009 (10)	0.0014 (9)	−0.0010 (9)
C9	0.0600 (18)	0.120 (3)	0.0375 (13)	0.0260 (16)	−0.0047 (12)	−0.0161 (14)
C10	0.0616 (19)	0.127 (3)	0.0485 (15)	0.0296 (18)	0.0082 (14)	−0.0186 (16)
C11	0.0414 (13)	0.0509 (14)	0.0566 (14)	0.0011 (11)	0.0053 (11)	−0.0013 (11)
C12	0.0492 (16)	0.101 (2)	0.0461 (14)	0.0154 (14)	−0.0064 (12)	0.0001 (14)
C13	0.0492 (15)	0.095 (2)	0.0355 (13)	0.0157 (14)	0.0000 (11)	−0.0048 (12)
C14	0.0482 (16)	0.081 (2)	0.0845 (19)	0.0108 (14)	0.0077 (14)	−0.0042 (16)
N1	0.0414 (11)	0.0676 (14)	0.0297 (10)	0.0122 (9)	−0.0026 (8)	−0.0012 (9)
O1	0.0541 (10)	0.0812 (12)	0.0291 (8)	0.0129 (8)	−0.0065 (7)	−0.0015 (7)
Cl1	0.0697 (5)	0.0710 (5)	0.0924 (5)	0.0008 (4)	−0.0054 (4)	0.0200 (4)
Cl2	0.0594 (5)	0.0703 (5)	0.0853 (5)	−0.0040 (3)	0.0068 (3)	0.0012 (4)

### Geometric parameters (Å, °)

**Table:**

C1—C2	1.383 (3)	C8—C9	1.371 (3)
C1—C6	1.386 (3)	C8—C13	1.371 (3)
C1—N1	1.417 (3)	C9—C10	1.368 (4)
C2—C3	1.377 (3)	C9—H9	0.9300
C2—Cl1	1.731 (3)	C10—C11	1.357 (3)
C3—C4	1.374 (4)	C10—H10	0.9300
C3—H3	0.9300	C11—C12	1.370 (3)
C4—C5	1.364 (4)	C11—C14	1.504 (3)
C4—H4	0.9300	C12—C13	1.373 (3)
C5—C6	1.377 (3)	C12—H12	0.9300
C5—H5	0.9300	C13—H13	0.9300
C6—Cl2	1.727 (2)	C14—H14A	0.9600
C7—O1	1.229 (2)	C14—H14B	0.9600
C7—N1	1.350 (3)	C14—H14C	0.9600
C7—C8	1.484 (3)	N1—H1N	0.823 (16)
			
C2—C1—C6	117.5 (2)	C10—C9—C8	121.1 (2)
C2—C1—N1	121.5 (2)	C10—C9—H9	119.4
C6—C1—N1	121.0 (2)	C8—C9—H9	119.4
C3—C2—C1	121.9 (3)	C11—C10—C9	122.4 (2)
C3—C2—Cl1	118.8 (2)	C11—C10—H10	118.8
C1—C2—Cl1	119.30 (19)	C9—C10—H10	118.8
C4—C3—C2	118.8 (3)	C10—C11—C12	116.5 (2)
C4—C3—H3	120.6	C10—C11—C14	122.7 (2)
C2—C3—H3	120.6	C12—C11—C14	120.8 (2)
C5—C4—C3	121.0 (3)	C11—C12—C13	121.8 (2)
C5—C4—H4	119.5	C11—C12—H12	119.1
C3—C4—H4	119.5	C13—C12—H12	119.1
C4—C5—C6	119.6 (3)	C8—C13—C12	121.1 (2)
C4—C5—H5	120.2	C8—C13—H13	119.4
C6—C5—H5	120.2	C12—C13—H13	119.4
C5—C6—C1	121.3 (2)	C11—C14—H14A	109.5
C5—C6—Cl2	118.9 (2)	C11—C14—H14B	109.5
C1—C6—Cl2	119.88 (17)	H14A—C14—H14B	109.5
O1—C7—N1	120.3 (2)	C11—C14—H14C	109.5
O1—C7—C8	122.21 (18)	H14A—C14—H14C	109.5
N1—C7—C8	117.44 (17)	H14B—C14—H14C	109.5
C9—C8—C13	117.0 (2)	C7—N1—C1	121.85 (17)
C9—C8—C7	119.24 (19)	C7—N1—H1N	120.9 (17)
C13—C8—C7	123.73 (19)	C1—N1—H1N	117.2 (17)
			
C6—C1—C2—C3	0.3 (4)	O1—C7—C8—C13	174.2 (2)
N1—C1—C2—C3	−179.9 (2)	N1—C7—C8—C13	−5.5 (3)
C6—C1—C2—Cl1	178.38 (16)	C13—C8—C9—C10	0.7 (4)
N1—C1—C2—Cl1	−1.8 (3)	C7—C8—C9—C10	178.2 (3)
C1—C2—C3—C4	0.9 (4)	C8—C9—C10—C11	0.3 (5)
Cl1—C2—C3—C4	−177.2 (2)	C9—C10—C11—C12	−1.3 (5)
C2—C3—C4—C5	−1.0 (4)	C9—C10—C11—C14	−179.8 (3)
C3—C4—C5—C6	−0.1 (4)	C10—C11—C12—C13	1.3 (4)
C4—C5—C6—C1	1.4 (4)	C14—C11—C12—C13	179.9 (3)
C4—C5—C6—Cl2	−178.2 (2)	C9—C8—C13—C12	−0.6 (4)
C2—C1—C6—C5	−1.5 (3)	C7—C8—C13—C12	−178.0 (2)
N1—C1—C6—C5	178.7 (2)	C11—C12—C13—C8	−0.4 (4)
C2—C1—C6—Cl2	178.09 (17)	O1—C7—N1—C1	−2.5 (3)
N1—C1—C6—Cl2	−1.7 (3)	C8—C7—N1—C1	177.2 (2)
O1—C7—C8—C9	−3.2 (4)	C2—C1—N1—C7	105.8 (3)
N1—C7—C8—C9	177.2 (2)	C6—C1—N1—C7	−74.5 (3)

### Hydrogen-bond geometry (Å, °)

**Table:**

*D*—H···*A*	*D*—H	H···*A*	*D*···*A*	*D*—H···*A*
N1—H1N···O1^i^	0.82 (2)	2.08 (2)	2.878 (2)	164 (2)

Symmetry codes: (i) −*y*+3/4, *x*+1/4, *z*+1/4.
